# 3-Cyano­anilinium nitrate

**DOI:** 10.1107/S1600536809034886

**Published:** 2009-09-09

**Authors:** Bo Wang

**Affiliations:** a Ordered Matter Science Research Center, College of Chemistry and Chemical Engineering, Southeast University, Nanjing 210096, People’s Republic of China

## Abstract

In the cation of the title compound, C_7_H_7_N_2_^+^·NO_3_^−^, the nitrile group and the benzene ring are almost coplanar (r.m.s. deviation = 0.006 Å).  In the crystal, the ions are connected by bifurcated N—H⋯(O,O) hydrogen bonds, forming a two-dimensional network parallel to (001).

## Related literature

For the applications of metal-organic coordination compounds, see: Fu *et al.* (2007 ▶); Chen *et al.* (2001 ▶); Fu & Xiong (2008 ▶); Xiong *et al.* (1999 ▶); Xie *et al.* (2003 ▶); Zhao *et al.* (2004 ▶). For nitrile derivatives, see: Fu *et al.* (2008 ▶); Wang *et al.* 2002 ▶.
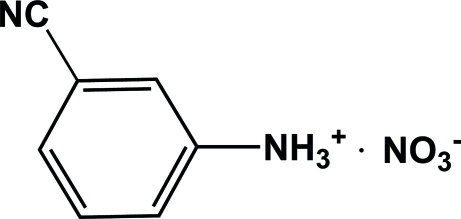


## Experimental

### 

#### Crystal data

C_7_H_7_N_2_^+^·NO_3_^−^
*M* *_r_* = 181.16
Orthorhombic, 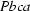

*a* = 10.210 (2) Å
*b* = 10.812 (2) Å
*c* = 15.398 (3) Å
*V* = 1699.8 (6) Å^3^
*Z* = 8
Mo *K*α radiation
μ = 0.11 mm^−1^
*T* = 298 K
0.40 × 0.25 × 0.20 mm

#### Data collection

Rigaku Mercury2 diffractometer
Absorption correction: multi-scan (*CrystalClear*; Rigaku, 2005 ▶) *T*_min_ = 0.94, *T*_max_ = 1.00
15905 measured reflections
1871 independent reflections
1456 reflections with *I* > 2σ(*I*)
*R*_int_ = 0.062

#### Refinement

*R*[*F* ^2^ > 2σ(*F* ^2^)] = 0.052
*wR*(*F* ^2^) = 0.141
*S* = 1.14
1871 reflections
120 parameters
H-atom parameters constrained
Δρ_max_ = 0.21 e Å^−3^
Δρ_min_ = −0.19 e Å^−3^

### 

Data collection: *CrystalClear* (Rigaku, 2005 ▶); cell refinement: *CrystalClear*; data reduction: *CrystalClear*; program(s) used to solve structure: *SHELXS97* (Sheldrick, 2008 ▶); program(s) used to refine structure: *SHELXL97* (Sheldrick, 2008 ▶); molecular graphics: *SHELXTL* (Sheldrick, 2008 ▶); software used to prepare material for publication: *SHELXTL*.

## Supplementary Material

Crystal structure: contains datablocks I, global. DOI: 10.1107/S1600536809034886/ci2892sup1.cif

Structure factors: contains datablocks I. DOI: 10.1107/S1600536809034886/ci2892Isup2.hkl

Additional supplementary materials:  crystallographic information; 3D view; checkCIF report

## Figures and Tables

**Table 1:** Hydrogen-bond geometry (Å, °)

*D*—H⋯*A*	*D*—H	H⋯*A*	*D*⋯*A*	*D*—H⋯*A*
N2—H2*A*⋯O3^i^	0.89	2.22	3.104 (2)	173
N2—H2*A*⋯O1^i^	0.89	2.44	3.107 (2)	133
N2—H2*B*⋯O2^ii^	0.89	2.06	2.859 (2)	150
N2—H2*B*⋯O3^ii^	0.89	2.25	3.049 (2)	149
N2—H2*C*⋯O2	0.89	1.85	2.738 (2)	172
N2—H2*C*⋯O1	0.89	2.56	3.090 (2)	119

Symmetry codes: (i) 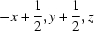; (ii) 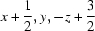.

**Fig. 1.**
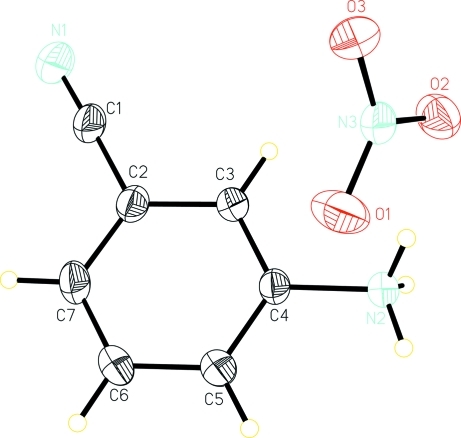
A view of the title compound with the atomic numbering scheme. Displacement ellipsoids are drawn at the 30% probability level.

**Fig. 2.**
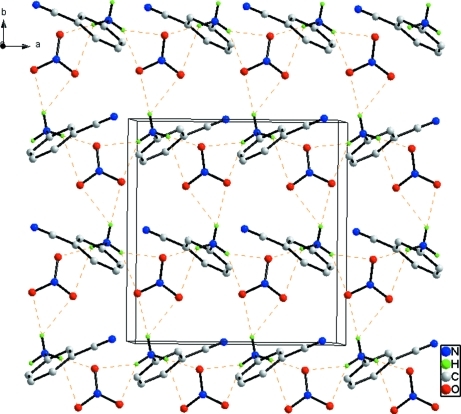
The crystal packing of the title compound, viewed along the c axis, showing N—H···O hydrogen bonds (dashed lines). H atoms not involved in hydrogen bonding have been omitted for clarity.

## supplementary crystallographic information

### Comment

The construction of metal-organic coordination compounds has attracted much attention owing to potential functions, such as permittivity, fluorescence, magnetism and optical properties (Fu *et al.*, 2007; Chen *et al.*, 2001; Fu & Xiong, 2008; Xie *et al.*, 2003; Zhao *et al.*, 2004; Xiong *et al.*, 1999). Nitrile derivatives are a class of excellent ligands for the construction of novel metal-organic frameworks (Wang *et al.* 2002; Fu *et al.*, 2008). We report here the crystal structure of the title compound, 3-cyanoanilinium nitrate.

In the 3-cyanoanilinium cation (Fig.1), the nitrile group and the benzene ring are coplanar. The nitrile group C1≡N1 bond length of 1.102 (3) Å is within the normal range.

In the crystal structure, all the amine group H atoms are involved in N—H···O hydrogen bonds (Table 1) with O atoms of the NO_3_^-^ anion. These hydrogen bonds link the ionic units into a two-dimensional network (Fig. 2) parallel to the (001) plane.

### Experimental

The commercial 3-aminobenzonitrile (3 mmol, 0.55 g) and HNO_3_ (0.5 ml) were dissolved in ethanol (20 ml). Colourless block-shaped crystals of the title compound suitable for X-ray analysis were obtained by slow evaporation at room temperature.

### Refinement

H atoms were positioned geometrically and treated as riding, with C-H = 0.93 Å, N-H = 0.89 Å and *U*_iso_(H) = 1.2U_eq_(C) and *U*_iso_(H) = 1.5U_eq_(N). A rotating-group model was used for the -NH_3_ group.

### Crystal data

**Table:**

C_7_H_7_N_2_^+^·NO_3_^−^	*F*(000) = 752
*M**_r_* = 181.16	*D*_x_ = 1.416 Mg m^−^^3^
Orthorhombic, *P**b**c**a*	Mo *K*α radiation, λ = 0.71073 Å
Hall symbol: -P 2ac 2ab	Cell parameters from 1456 reflections
*a* = 10.210 (2) Å	θ = 3.1–27.5°
*b* = 10.812 (2) Å	µ = 0.11 mm^−^^1^
*c* = 15.398 (3) Å	*T* = 298 K
*V* = 1699.8 (6) Å^3^	Block, colourless
*Z* = 8	0.40 × 0.25 × 0.20 mm

### Data collection

**Table:**

Rigaku Mercury2 diffractometer	1871 independent reflections
Radiation source: fine-focus sealed tube	1456 reflections with *I* > 2σ(*I*)
graphite	*R*_int_ = 0.062
Detector resolution: 13.6612 pixels mm^-1^	θ_max_ = 27.5°, θ_min_ = 3.1°
CCD profile fitting scans	*h* = −13→13
Absorption correction: multi-scan (*CrystalClear*; Rigaku, 2005)	*k* = −13→13
*T*_min_ = 0.94, *T*_max_ = 1.00	*l* = −19→19
15905 measured reflections	

### Refinement

**Table:**

Refinement on *F*^2^	Secondary atom site location: difference Fourier map
Least-squares matrix: full	Hydrogen site location: inferred from neighbouring sites
*R*[*F*^2^ > 2σ(*F*^2^)] = 0.052	H-atom parameters constrained
*wR*(*F*^2^) = 0.141	*w* = 1/[σ^2^(*F*_o_^2^) + (0.059*P*)^2^ + 0.3685*P*] where *P* = (*F*_o_^2^ + 2*F*_c_^2^)/3
*S* = 1.14	(Δ/σ)_max_ = 0.001
1871 reflections	Δρ_max_ = 0.21 e Å^−^^3^
120 parameters	Δρ_min_ = −0.19 e Å^−^^3^
0 restraints	Extinction correction: *SHELXL97* (Sheldrick, 2008), Fc^*^=kFc[1+0.001xFc^2^λ^3^/sin(2θ)]^-1/4^
Primary atom site location: structure-invariant direct methods	Extinction coefficient: 0.021 (3)

### Special details

**Table:**

Geometry. All esds (except the esd in the dihedral angle between two l.s. planes) are estimated using the full covariance matrix. The cell esds are taken into account individually in the estimation of esds in distances, angles and torsion angles; correlations between esds in cell parameters are only used when they are defined by crystal symmetry. An approximate (isotropic) treatment of cell esds is used for estimating esds involving l.s. planes.
Refinement. Refinement of F^2^ against ALL reflections. The weighted R-factor wR and goodness of fit S are based on F^2^, conventional R-factors R are based on F, with F set to zero for negative F^2^. The threshold expression of F^2^ > 2sigma(F^2^) is used only for calculating R-factors(gt) etc. and is not relevant to the choice of reflections for refinement. R-factors based on F^2^ are statistically about twice as large as those based on F, and R- factors based on ALL data will be even larger.

### Fractional atomic coordinates and isotropic or equivalent isotropic displacement parameters (Å^2^)

**Table:**

	*x*	*y*	*z*	*U*_iso_*/*U*_eq_	
N1	0.06292 (19)	0.5067 (2)	0.39061 (13)	0.0724 (6)	
N2	0.39497 (14)	0.44355 (16)	0.69503 (9)	0.0435 (4)	
H2A	0.4110	0.5239	0.7016	0.065*	
H2B	0.4620	0.4001	0.7158	0.065*	
H2C	0.3224	0.4236	0.7238	0.065*	
C1	0.1482 (2)	0.4737 (2)	0.42791 (13)	0.0526 (5)	
C2	0.25733 (17)	0.43017 (19)	0.47337 (11)	0.0443 (5)	
C3	0.27221 (17)	0.45958 (18)	0.56150 (11)	0.0420 (5)	
H3	0.2108	0.5080	0.5904	0.050*	
C4	0.37764 (16)	0.41559 (17)	0.60241 (11)	0.0382 (4)	
C5	0.46585 (19)	0.34385 (19)	0.55934 (12)	0.0488 (5)	
H5	0.5382	0.3131	0.5890	0.059*	
C6	0.4502 (2)	0.3155 (2)	0.47200 (13)	0.0568 (6)	
H6	0.5119	0.2669	0.4436	0.068*	
C7	0.3461 (2)	0.3585 (2)	0.42905 (12)	0.0538 (5)	
H7	0.3339	0.3403	0.3706	0.065*	
O1	0.23542 (15)	0.20195 (16)	0.71257 (11)	0.0701 (5)	
O2	0.16047 (13)	0.37804 (14)	0.76798 (10)	0.0594 (5)	
O3	0.04000 (13)	0.21817 (14)	0.73494 (10)	0.0592 (5)	
N3	0.14567 (15)	0.26571 (16)	0.73782 (10)	0.0449 (4)	

### Atomic displacement parameters (Å^2^)

**Table:**

	*U*^11^	*U*^22^	*U*^33^	*U*^12^	*U*^13^	*U*^23^
N1	0.0625 (12)	0.1071 (17)	0.0476 (11)	0.0072 (12)	−0.0138 (9)	−0.0010 (11)
N2	0.0413 (8)	0.0563 (10)	0.0328 (8)	0.0053 (7)	−0.0013 (6)	−0.0047 (7)
C1	0.0517 (11)	0.0722 (14)	0.0339 (10)	−0.0038 (10)	−0.0043 (9)	−0.0017 (9)
C2	0.0432 (10)	0.0547 (12)	0.0351 (9)	−0.0056 (8)	−0.0034 (7)	0.0006 (8)
C3	0.0382 (9)	0.0536 (11)	0.0341 (9)	0.0008 (8)	0.0015 (7)	−0.0030 (8)
C4	0.0401 (9)	0.0440 (10)	0.0306 (9)	−0.0018 (8)	0.0004 (7)	−0.0021 (7)
C5	0.0473 (10)	0.0588 (12)	0.0403 (10)	0.0103 (9)	0.0008 (8)	−0.0038 (9)
C6	0.0572 (13)	0.0703 (14)	0.0429 (11)	0.0126 (11)	0.0061 (9)	−0.0134 (10)
C7	0.0612 (12)	0.0685 (14)	0.0318 (9)	−0.0020 (11)	0.0013 (9)	−0.0094 (9)
O1	0.0487 (9)	0.0727 (11)	0.0889 (12)	0.0073 (8)	0.0163 (8)	−0.0161 (9)
O2	0.0548 (9)	0.0635 (10)	0.0598 (9)	−0.0069 (7)	0.0087 (7)	−0.0173 (8)
O3	0.0395 (8)	0.0752 (10)	0.0628 (10)	−0.0077 (7)	−0.0046 (6)	−0.0065 (7)
N3	0.0440 (9)	0.0596 (11)	0.0310 (8)	−0.0007 (8)	−0.0012 (6)	0.0000 (7)

### Geometric parameters (Å, °)

**Table:**

N1—C1	1.102 (3)	C4—C5	1.361 (3)
N2—C4	1.469 (2)	C5—C6	1.389 (3)
N2—H2A	0.89	C5—H5	0.93
N2—H2B	0.89	C6—C7	1.335 (3)
N2—H2C	0.89	C6—H6	0.93
C1—C2	1.397 (3)	C7—H7	0.93
C2—C7	1.374 (3)	O1—N3	1.211 (2)
C2—C3	1.402 (3)	O2—N3	1.309 (2)
C3—C4	1.335 (2)	O3—N3	1.196 (2)
C3—H3	0.93		
			
C4—N2—H2A	109.5	C3—C4—N2	118.82 (15)
C4—N2—H2B	109.5	C5—C4—N2	120.72 (16)
H2A—N2—H2B	109.5	C4—C5—C6	121.42 (18)
C4—N2—H2C	109.5	C4—C5—H5	119.3
H2A—N2—H2C	109.5	C6—C5—H5	119.3
H2B—N2—H2C	109.5	C7—C6—C5	119.66 (19)
N1—C1—C2	178.6 (2)	C7—C6—H6	120.2
C7—C2—C1	117.84 (17)	C5—C6—H6	120.2
C7—C2—C3	122.50 (17)	C6—C7—C2	118.37 (17)
C1—C2—C3	119.66 (17)	C6—C7—H7	120.8
C4—C3—C2	117.60 (17)	C2—C7—H7	120.8
C4—C3—H3	121.2	O3—N3—O1	115.22 (17)
C2—C3—H3	121.2	O3—N3—O2	121.07 (16)
C3—C4—C5	120.45 (17)	O1—N3—O2	123.69 (16)

### Hydrogen-bond geometry (Å, °)

**Table:**

*D*—H···*A*	*D*—H	H···*A*	*D*···*A*	*D*—H···*A*
N2—H2A···O3^i^	0.89	2.22	3.104 (2)	173
N2—H2A···O1^i^	0.89	2.44	3.107 (2)	133
N2—H2B···O2^ii^	0.89	2.06	2.859 (2)	150
N2—H2B···O3^ii^	0.89	2.25	3.049 (2)	149
N2—H2C···O2	0.89	1.85	2.738 (2)	172
N2—H2C···O1	0.89	2.56	3.090 (2)	119

Symmetry codes: (i) −*x*+1/2, *y*+1/2, *z*; (ii) *x*+1/2, *y*, −*z*+3/2.
